# Deaths under Anæsthetics: The Relation of Coroners and Hospital Staffs

**Published:** 1915-02-13

**Authors:** 


### DEATHS UNDER ANÆSTHETICS.

### The Relation of Coroners and Hospital Staffs.

An inquest recently held at Chelsea raises two points which are of importance both to the medical profession and to those charged with the administration of hospitals. The facts are briefly that a child suffering from osteo-myelitis was operated on in the usual fashion in the Cheyne Hospital; that it did not take the anæsthetic well; that after some degree of recovery it was taken back to the ward, where it shortly became unconscious and died before medical assistance could be obtained. By this time the surgeon and the anæsthetist had left the hospital, and the institution, it appears, has no resident medical officer. Subsequently the surgeon gave a certificate of death, but the facts coming to the attention of the Coroner an inquest was held.

The first point to consider is whether in hospitals having no resident medical officer the performance of serious operations needing a general anæsthetic should be sanctioned, unless some provision is made for medical attendance until the patient's recovery has reached a point of safety. The large success attending the use of anæsthetics, and the local benefits due to such hospitals, must not blind us to the risk of such incidents as here reported. No one can say that assistance would have saved this unfortunate child, but it ought to have had what help was possible; and it is clearly a responsibility which cannot be avoided that a medical man who anæsthetises a patient shall see, either directly or by deputy, that until the effect of the drug has disappeared adequate medical assistance shall be at hand. If this cannot be secured, then, unless in some acute emergency, anæsthesia ought not to be undertaken.

### The Surgeon's Defence.

The second issue raised by the case refers to the granting of the death certificate. The surgeon defended his action in this respect by the plea that the child did not die during the operation and that the unconsciousness which preceded death was not due to the anæsthetic. Apparently he did not recognise that there was an issue altogether preliminary to the operation. Admittedly the child was suffering from osteo-myelitis; and, to put the argument in the mildest form, the osteo-myelitis may have been due to an injury. Indeed, we fancy that most surgeons would say that some form of injury must have occurred. But this alone would bring the death within the jurisdiction of the Coroner, who has authority to act whenever there is any reasonable ground to suspect that violence has been one of the factors responsible for the fatal issue.

Although the violence may have been remote and may, in the judgment of the practitioner, have played no part in the causation of the death, it still remains true that the very fact of its occurrence is sufficient to demand that the case be reported to the Coroner. It is for his jury, not for the practitioner, to decide what contribution (if any) violence has made to the fatal event. This may seem ridiculous to the medical profession, and perhaps it is ridiculous. All the same it is the law of the land, and all good citizens will therefore obey it. Doctors must be reminded of this claim, if necessary with wearisome iteration. Each man may hold what individual opinion he will, but his action must be determined not by his own judgment, but by the rules prescribed by the Legislature.

### The Coroner's Inquiries.

In the present instance, therefore, the existence of osteo-myelitis, with the necessary presumption of injury or violence, was sufficient to justify some inquiries by the Coroner. There was, or there was possibly, an element of violence in the case. Hence, the surgeon ought either to have communicated directly with the Coroner or to have refused a certificate of death. All that legally could be demanded of him was to have taken the latter course. The obligation on a practitioner is either to give a death certificate or to refuse it. Refusal, no doubt, will mean the intervention of the Coroner, as without the death certificate the death cannot be registered, and without registration the body cannot be buried.

But with all this, it is to be noted, the practitioner, strictly speaking, is not concerned. What follows the refusal of the death certificate is determined by law, and not by the practitioner. At the same time may it not be advisable, when any doubt arises, for the medical attendant to communicate the facts privately to the Coroner, and in this way to learn whether the Coroner thinks the facts do or do not demand an inquest? A good deal of private ill-feeling and open and unlovely contention would be spared were this course adopted. There have no doubt been Coroners with whom friendly relations have been impossible, and the only thing for these gentry is to hold them to the strict letter of the law. But, speaking generally, a little tact and reasonable recognition of official responsibility will secure good relations and smooth working for all concerned.

The same policy applies in relation to deaths during or shortly after operations. If it is well to communicate with the Coroner regarding the first of these classes, it is well also in relation to the second. In the present instance, the surgeon concerned established a distinction between the two classes, and said that one of the large London hospitals acted on this distinction. Whether this is so or not we cannot say, but we feel strongly that the wise and commonsense course is for doctors and hospitals on the one hand, and for Coroners on the other, to cultivate open, frank, and friendly relations, and not to develop a rigid and unbending etiquette. The demand for commonsense is in both directions, and failure has not always been on the medical side. In the present instance we think the surgeon was wrong, and it is in the public interest that the causes of the blunder should be recorded.

